# Navigating Cardiotoxicity in Cancer Treatment: Insights into Fluoropyrimidine-induced Cardiac Events

**DOI:** 10.2174/011573403X335593250413154832

**Published:** 2025-04-28

**Authors:** Ashika Bhattarai, Manodeep Chakraborty, Md. Hasnat Jahan Ali, Chetiz Sharma, Akanchya Rai, Rudra Acharya, Yuan Rai, Ananya Bhattacharjee, Nihar Ranjan Bhuyan

**Affiliations:** 1 Department of Pharmacology, Himalayan Pharmacy Institute, Majhitar, Rangpo, East Sikkim, India

**Keywords:** Fluropyrimidine, 5-FU, cardiotoxicity, coronary vasospasm, myocarditis, DPYD gene

## Abstract

**Introduction:**

Fluoropyrimidine (FP) is a key cancer treatment but often causes side effects, notably cardiotoxicity. This cardiotoxicity can present as angina, arrhythmia, dyspnea, and palpitations, requiring urgent cardiologist attention. The etiology, management, and frequency of FP-induced cardiotoxicity are still unknown despite long-term use.

**Objective:**

The article aims to provide an overview of the pathogenic occurrence, cardiac event risk factor, possible underlying mechanism of FP-cardiotoxicity, diagnostics, and therapeutic approach for the corrective management of this clinical condition.

**Methods:**

Review was performed by searching extensively for various existing literature search PubMed, Web of science and Scopus using suitable keywords to find articles that support our review study.

**Results and Discussion:**

FP induced cardiotoxicity results in morbidness and fatality in patient undergoing the treatment. Thus, an effective management system must be standardized to effectively treat and prevent this clinical condition.

**Conclusion:**

The cardiotoxic event following 5-FU has been lesser-known clinical entity with limited study on its pathophysiology and management. In the diagnosis procedure, each patient undergoing FP treatment ought to have early symptom identity, risk categorization, and therapy individualized based on benefit-risk ratio.

## 1 INTRODUCTION

Cancer in modern day has proven to be the most vicious diseases that is accountable for a large no of mortality every year with an estimation of more than 19.3 million new incidents and about 10 million deaths as reported in GLOBOCAN 2020 [1]. Globalization and threats related with growing economies may exacerbate this, but it is anticipated that the number of cancer cases worldwide will rise to 28.4 million in next 40 years, a 47 percent increase over 2020. The increase in transition countries (64% to 95%) compared to non-transition countries (32% to 56%) will be primarily driven by demographic changes [2]. This alarming rise in the cases of this disease requests for newer intervention and use of more potent treatment regimes. Use of various chemotherapeutic drugs and radiotherapy still provides as primary treatment measures but they always come with side effects that would potentially damage patient wellness and vitality [3]. Therefore, it is crucial to understand the causes and develop innovative approaches to mitigate the side effects associated with chemotherapy.

### 1.1 Fluoropyrimidines in Cancer Treatment

Fluoropyrimidines, including 5-fluorouracil and capecitabine, have been implemented in medicine as chemotherapy agents since their discovery. They are employed to treat cancers originating from glandular and squamous cells, such as those located in the craniofacial region, gastrointestinal tract, bladder, pancreas, and breast [4]. Its application is frequently regarded as a conventional approach in the handling of advanced colorectal cancer. This is attributed to its demonstrated synergistic impact when combined with external beam radiation, enhancing the tumor's responsiveness to the radiation therapy [4, 5].

5-FU, a pyrimidine derivative, exerts cytotoxic effects on cancer cells by inhibiting thymidylate synthase (TS), an enzyme essential for DNA replication. As S-phase antimetabolites, these agents induce genomic instability by causing double-strand and single-strand DNA breaks, disrupting DNA synthesis, repair, and elongation [6]. The enzymes thymidine phosphorylase and cytidine deaminase are engaged in a sequence of events that convert capecitabine into 5-FU. Since these enzymes are upregulated in cancer cells, they preferentially target malignant tissues over regularly dividing ones [7]. However, like other chemotherapy drugs, the potential advantages of fluoropyrimidines must be carefully considered in relation to the drug-associated risks and toxicities. Among the diverse side effects such as dose-limiting hand-foot syndrome, mucositis, and diarrhea, dose limiting myelosuppression especially in individuals afflicted by DPD inadequacy or those who experience a drug overdose can reach elevated concentrations of 5FU leading to heart problems, inflammation of the colon, decreased WBC count, and brain dysfunction [8]. It is important to emphasize the significance of cardiotoxicity and hepatotoxicity in these cases. The antitumor effects of fluoropyrimidines have been thoroughly investigated and comprehended, but the mechanisms behind fluoropyrimidine-induced heart and liver toxicity remain to be elucidated.

### 1.2 5-FU Induced Cardiotoxicity

Cardiotoxicity is acknowledged as a rare yet impactful adverse effect, within the realm of conventional chemotherapeutic drugs, 5-FU emerges as the second highest prominent contributor to chemotherapy-induced cardiotoxicity, trailing only behind anthracyclines [5]. Coronary artery spasm-induced angina and coronary artery thrombosis-induced IHD are two cardiovascular problems associated with fluoropyrimidines, particularly 5-FU. There have also there have been direct observations of cardiac injury, which can result in further critical heart toxicity. This may show up as shock, arrhythmia, cardiomyopathy, heart failure, hypertension, hypotension, shock, unexpected heart failure risk, to mention some medical conditions [5, 9-11]. The necessity for effective interventions addressing cardiovascular diseases (CVD) in both individuals with cancer and their survivors has led to growth of the medical subspecialty known as cardio-oncology [10].

### 1.3 5-FU Induced Pathological Incidence

The reported incidence rates range from 1.2% to 18% among exposed patients [12] and significantly contributes to heightened mortality and morbidity rates of 2.2-13.3% [13-15]. The prevalence of cardiac toxicity in association with Fluoropyrimidines depends largely on the route through which the drug has been administered. In accordance to cardiotoxicity attributable to 5-FU, it has been observed that cardiotoxicity occurs most commonly when the drug is administered *via* a continuous intravenous infusion [10, 11, 16, 17]. The incidence rates of cardiotoxicity linked to various fluoropyrimidine treatment regimens used in treating different types of cancer is demonstrated in Table **1**.

The initial symptoms of this phenomenon are reported to appear at any point during the infusion, within 12 hours after the initial intravenous administration, or at any time within 1-2 days thereafter [5, 18]. Based on a different study, the first signs usually appear three or four days into a five-day continuous infusion and after the fourth intravenous bolus therapy [19, 20], thus, indicating that individuals undergoing infusion-based treatment plans exhibit a greater susceptibility compared to those undergoing bolus regimens [12, 21]. The overview of research assessing the frequency of cardiotoxicity in patients receiving 5-FU treatment is give in Table **2** [17, 23-32].

### 1.4 Clinical Manifestation and Risk Factor

Angina stands out as the predominant sign of fluoropyrimidine-induced cardiotoxicity (FIC), occurring in approximately 19–45% of patients, regardless of the manifestation of ST and T wave changes on electrocardiograms (ECGs) [33, 34]. Several prospective and retrospective studies have demonstrated that the prevalent symptoms of cardiotoxicity brought on by fluoropyrimidine (FP) administration encompass myocardial infarction (MI), heart failure, myopericarditis, arrhythmias, QT prolongation, and cardiogenic shock (requiring extracorporeal membrane oxygenation support and an intra-aortic balloon pump) [35, 36] and heart arrest [11, 36]. Besides symptomatic effect of cardiotoxicity, some patients may experience silent cardiac ischemia, along with symptoms like palpitations, dyspnea, and pleuritic chest pain, occurring either at rest or during physical exertion [24]. Notably, there appears to be no distinction between cardiovascular toxicities induced by 5-FU and those induced by capecitabine [37]. Reported associated arrhythmias comprise bradycardia, atrial fibrillation, ventricular tachycardia, and ventricular fibrillation [38]. Furthermore, there are also existed documented instances of takotsubo cardiomyopathy following FP administration [39].

Cardiovascular adverse effects linked to FPs usually appears through the initial cycle of treatment, usually 72 hours after injection [40]. In a retrospective case-control study by Raber *et al.,* anginal symptoms were the prevalent sign of cardiotoxicity, appearing in every case of 177 patients administered with either capecitabine or 5-fluorouracil (5-FU). Among these, two patients (constituting 25% of cases) exhibited new-onset left ventricular dysfunction, as evidenced by comparison with previous echocardiograms, although baseline echocardiography data were not available for the entire cohort [28]. In another study conducted by Saif *et al.,* which involved 377 patients experiencing cardiac damage due to fluoropyrimidines, angina was the most common symptom, occurring in 45% of cases. Heart failure contributed 2%, myocardial infarction contributed 22%, arrhythmias contributed 23%, cardiac arrest contributed 1.4%, and acute pulmonary oedema contributed 5% [33].

The varying incidence rates of disease could be linked to divergences in risk profiles among patient groups and variations in drug administration schedules [10]. A meta-analysis encompassing 22 published observational studies (two pooled analyses of RCTs, one case-control, and 19 cohort studies) divided risk variables associated with FIC into two distinct groups: treatment-related factors and patient-related one [41].

Risk factors linked to the patient included a broad range of traits including age, gender, race, Eastern Cooperative Oncology Group (ECOG) score, quantity of metastases, location, size, and grade of the tumor, heart disease determinants, pre-existing cardiac conditions, hypertension, hyperlipidemia, hypercholesterolemia, stroke, anemia, neutropenia, infections, diabetes mellitus, renal and thyroid diseases, body mass index (BMI), smoking, and alcohol intake [29, 42, 43]. On the other hand, treatment-related risk factors included various chemotherapy regimens (such as those containing 5-FU either alone or in combination, including capecitabine-based treatments), and combinations of 5-FU with other agents like leucovorin, cisplatin, mitomycin, or cyclophosphamide. The meta-analysis identified several patient factors, with history of cardiac ailment present in 29% of the studies. Hypertension was identified in 12%, hyperglycemia and hypercholesterolemia each in 6%, older age in 12%, smoking in 12%, and female gender in 6% of the included studies. Concerning treatment-related factors, continuous infusion was observed in 24% of instances, while concurrent leucovorin or cisplatin administration was responsible for 18% of cases. Furthermore, 12% of instances were linked to capecitabine-based chemotherapy, 12% to high-dose 5-fluorouracil, and 6% to concomitant radiation therapy [41].

In a different study examining 377 cases of fluoropyrimidine (FP)-related cardiotoxicity, researchers found that just 14% of patients had a record of heart disease, while 37% were identified as having known predisposing factors for heart ailment. Among these factors smoking is most foremost factor [33]. Moreover, cardiac diseases often have a propensity for recurrence or may lead to post-cardiac injury syndromes [44], particularly considering the potential cardiovascular harm caused by fluoropyrimidine usage [45]. It is worth noting that pre-existing cardiac conditions span a spectrum of cardiac ailments, each potentially posing a different risk for FIC. For example, Mayer *et al.* discovered that patients diagnosed with ischemic heart disease (IHD) faced the greatest risk of fluoropyrimidine-induced cardiotoxicity compared to those with other cardiac conditions such as prior myocardial infarction, cardiac arrhythmias, cardiomyopathy, or congestive heart failure [13]. Therefore, in accurately evaluating the likelihood of FIC in cancer patients, careful evaluation of the specific pre-existing cardiac condition is essential [41].

However, traditional cardiovascular disease risk factors might not play a significant role in FP pertaining cardiovascular toxicity induction [45]. Despite advanced age being associated with an increased risk, the evidence supporting this is not robust [22, 33, 46]. Overall, there is not ample of solid evidence to support risk stratification for discontinuing FP administration based on patient characteristics. The meta-analysis revealed that patient undergoing chemotherapy regime with history of cardiac illness were at over three times higher risk of FIC compared to those without such conditions [41]. It is reasonable to conclude that the findings suggest FP-related cardiovascular toxicity may be worsened by concurrent chest radiotherapy [47], multidrug chemotherapy [13], a preexisting case of CAD and structural heart abnormalities (*e.g.*, valvular disease, cardiomyopathies) [48, 49]. Additionally, it was found that combining fluoropyrimidine (FP) with cisplatin and leucovorin heightened the chances of cardiovascular damage associated with 5-FU [17, 34]. Notably, 5-FU is also acknowledged to have its use as radiosensitizer during radiation therapy, and investigations have indicated that it may accelerate small artery thrombosis and cause cardiovascular problems [23, 33].

Finally, certain individuals are occasionally genetically susceptible to 5-fluorouracil (5-FU) toxicity. A review study of genetic mutation revealed that patients carrying a triple repeat variant of the thymidylate synthase (TS) promoter exhibited heightened expression of TS, the main target of 5-FU [50]. While the manifestation of this homozygous variant among patients with colorectal cancer correlated with reduced response rates to 5-FU therapy, it was also linked to decreased incidence of 5-FU toxicity. Furthermore, reduced levels of DYPD, the principal biological catalyst accountable for metabolizing 5-FU, have been associated with an increased susceptibility to 5-FU toxicity [5].

### 1.5 Understanding Mechanisms Underlying Fluoropyrimidine Toxicity

The mechanism behind fluoropyrimidines induce heart damage remains elusive, even though several theories have been posited that includes constriction of the coronary arteries, direct harm to cardiac muscle cells, impaired operation of the blood vessel lining, and enhances the predisposition for blood clot formation. Among these, the most widely recognized mechanism is coronary artery spasm, which can result in sudden reduction of blood flow to the heart, supported by both laboratory and animal studies [11]. Other purposed mechanism includes direct myocardial injury, stimulation of autoimmune response [51], interference of Krebs cycle, positive ionotropic & chronotropic effect [33], failed oxygen delivery [11], endarteritis [52], & accelerated atherosclerosis [33, 53].

#### 1.5.1 Coronary Vasospasm

To gain a deeper understanding of the phenomena, Kanduri *et al.* conducted review research in which they further categorized the biological mechanism of FP-induced coronary vasospasm into two categories: endothelium dependent mechanism (endothelial dysfunction) and endothelial independent mechanism primary smooth muscle [5].

##### 1.5.1.1 Endothelial Dysfunction

Acts as the vasculature's unique, almost universal reaction to assortment of injuries and medical situations [21], marking the initial phase of atherosclerosis. Preclinical research indicates that this malfunction should cause both endothelial cells and myocytes to initiate the apoptotic and autophagic pathways [54]. An additionally consequence of vascular endothelial impairment is augmented in blood levels of vasoconstrictors such as urotensin-2 and endothelin-1, as shown in 5-FU-treated individuals [55]. Studies employing scanning electron microscopy on rabbits given 5-FU showed significant endothelial damage, platelet accumulation, and fibrin formation, suggesting that a thrombogenic effect may have begun after an artery was injured [56]. The underlying reason for the patients' treatment, which is tumors, intensifies the procoagulant effect even more [19]. In clinical scenarios, the identification of vascular endothelial impairment often hinges on irregular vasodilation in reaction to elevated flow (shear stress) or vasodilators that are reliant on the endothelium, like acetylcholine [57, 58]. Consequently, myocardial ischemia is caused by declined NO production, increased blood levels of endothelin-1 and von Willebrand factor, and platelet aggregation. These factors result in symptoms like angina pectoris and ischemic changes that is capable to be seen on electrocardiography (ECG) [23, 59].

##### 1.5.1.2 Endothelial-independent Dysfunction

Primarily involving smooth muscle resulting in vasoconstriction in a manner similar to atherosclerosis, but when the endothelium remains functionally intact, this can be evaluated through invasive pharmacological tests using nitroglycerin [60, 61]. Endothelial dysfunction, like atherosclerosis, is a broad-based illness that affects the coronary and peripheral arteries. It provides clinicians with a non-invasive way to evaluate endothelial function *via* flow-mediated dilation of the profunda brachii artery, and it demonstrates excellent agreement with invasive research on coronary endothelial function [62]. Therefore, it is anticipated that peripheral vasoconstriction brought on by 5-FU therapy will match coronary vasoconstriction; however, coronary angiography should confirm this directly. Furthermore, experimental laboratory-based studies conducted *in vitro* with the help of rabbit aorta rings exposed to different substances showed concentration-dependent vasoconstriction in reaction to 5-FU and retained vascular wall relaxation in feedback to ACh [15], indicating impaired primary smooth muscle function and preserved endothelial function [21]. This *in vitro* activation of kinase C (PK-C) mediated this endothelium-independent vasoconstriction [15, 63]. The administration of 5-FU treatment has been linked to heightened oxidative stress due to lipid peroxidation, the generation of reactive oxygen species, and decreased levels of glutathione. Cardiomyocytes are notably susceptible to oxidative damage from reactive oxygen species, largely due to their abundant mitochondrial presence [21, 64, 65]. Durak *et al.* illustrated that administering 5-FU to guinea pigs decreased superoxide dismutase and GPx activity while increasing catalase activity and malondialdehyde concentration [66]. Similarly, *in vitro*, 5-FU-treated cardiomyocytes exhibited an increase in oxidative stress [54]. Furthermore, research on animals demonstrated the critical function that 5-FU degradation plays in producing extremely toxic metabolites that is capable to upset the Krebs cycle [19]. Mechanism of cardiotoxicity due to 5-FU: coronary vasospasm because of direct impairment of the endothelium and primary smooth muscle dysfunction is represented in Fig. (**1**).

#### 1.5.2 Role of Endothelial NO Synthase, Endothelin-1, Protein Kinase C (PKC) and Acetylcholine in Pathogenesis of Coronary Vasospasm

##### 1.5.2.1 Endothelial Nitric Oxide Synthase (eNOS)

A proposed explanation for the pathophysiology of 5-FU-induced coronary spasm suggests that it damages the vascular endothelium *via* eNOS. This damage leads to coronary spasm and vasoconstriction independent of endothelial function, triggered by PKC activation [67]. The production of NO by eNOS, in conjunction with its actions on caveolin, calmodulin, and serine/threonine protein kinase Akt/PKB, is essential for controlling cardiovascular tone [68, 69]. Furthermore, in a rabbit model, PKC contributes to the vasoconstriction linked to 5-FU, resulting in the concentration-dependent constriction of vascular smooth muscle that is irrespective endothelial cells [63].

##### 1.5.2.2 Endothelin-1

Strong vasoconstrictor endothelin-1 produced by cardiac fibroblasts, cardiomyocytes, endothelial cells, and various other non-cardiac organs, including the lungs [70] is responsible to regulate myocardial capillary blood flow and coronary vascular resistance in diseases like CAD [71]. Factors like hypoxia, ischemia, or shear stress prompt vascular endothelial cells to produce and release endothelin-1, which originates from the precursor peptide known as big endothelin 1(ET-1) [72]. During the experiment conducted by Thyss *et al.* [73], it was observed that the patient receiving 5-FU especially those encountering cardiotoxicity had elevated level of ET-1 in their plasma supporting the purposed hypothesis of endothelin-1 being the suspect for 5-FU induced vasoconstriction [9]. Similarly, Salepci *et al.* [74], in its study observed patterns of increased level of endothelin-1 level in blood sample of patient undergoing 5-FU treatment, nevertheless this pattern was not just observed in patient experiencing vasoconstriction. Hence, to fully understand the role and effect of ET-1 in the arterial tone it is important to further investigate its level during 5-FU infusion in the patient [9, 75].

##### 1.5.2.3 Acetylcholine

NO released by endothelial cells as a reaction to acetylcholine, which causes vasodilation. This NO is absorbed by the smooth muscle lining the vessel, causing muscular relaxation and subsequent dilating of the channel *via* the cyclic guanosine monophosphate (cGMP) pathway. Any impairment to the endothelium cells interferes with this mechanism, which causes acetylcholine to cause a paradoxical vasoconstriction [57]. Consequently, chronic vasoconstriction related with 5-FU treatment may have cardiotoxic implications [75].

#### 1.5.3 Direct Myocardial Injury

Another pathway through which fluoropyrimidine cardiotoxicity manifests involves direct injury to myocardial cell, characterized by global impairment in systolic function that does not align with the perfusion territories of individual coronary arteries [16]. Following a ventricular biopsy conducted on rabbits, Kuropkat *et al.* identified the engagement of SR dilatation in this cardiotoxic process, a mechanism akin to the cardiac toxicity seen with anthracyclines [76]. Fluoroacetate and fluorocitrate, two metabolites of 5-FU, have seen to interfere with the Krebs cycle by hindering oxidative phosphorylation in cardiomyocytes. This results in enhanced citrate levels and ATP depletion [77]. This disruption in energy metabolism induces inflammatory cardiomyopathy and prompts apoptosis of cardiac myocytes [54]. The downstream breakdown product of 5-FU, alpha-fluoro-beta-alanine (FBAL), is identified as a key facilitator of this direct hazardous impact. TCA is competitively inhibited by fluorocitrate, which is produced when FBAL is transformed into fluoroacetate. As a result high-energy ATP is depleted, and citrate builds up that happen without reference to variations in the blood and oxygen supply [78, 79]. This is considered to be carried on by reduced aerobic efficiency primarily a result of mitochondrial uncoupling associated with 5-FU [21]. 5-FU treatment caused diminish level of ATP and citrate buildup in the ventricular myocardium, along with ischemia ECG changes in a guinea pig model, including ST segment elevation, depression, and T wave inversion [5, 80]. In a reported case, Muneoka *et al.* [78]. demonstrated elevated FBAL levels in a patient following 5-FU-induced myocardial infarction. Free radical damage is furthermore linked to the cardiotoxic effects of fluoropyrimidines. An *in vitro* investigation showed that cardiomyocytes and endothelial cells had elevated reactive oxygen species levels, which led to 5-FU-induced death [54]. Examination under electron microscopy showed damage to mitochondria, expansion of the endoplasmic reticulum, as well as existence of autophagic vacuoles. Furthermore, senescence-associated β-galactosidase activity increased, suggesting cellular senescence. Superoxide dismutase and GPx levels in the heart also decreased in guinea pigs given 5-FU, offering more proof that oxidative stress plays a part in this event [54, 66].

Some theories regarding the way to directly damage myocardial cells include Takotsubo cardiomyopathy, which is brought on by an overly sympathetic response [81], erythrocyte conversion to echinocytes, which raises blood viscosity and decreases oxygen-carrying capacity, resulting in ischemia [82], and vasospasm, which is brought on by an allergic reaction [11, 81]. Mechanism of 5-FU induced cardiotoxic event: myocarditis (direct myocardial cell damage) along with various factor contributing to the event is represented in Fig. (**2**).

#### 1.5.4 Other Proposed Mechanism

##### 1.5.4.1 Autoimmune Stimulation

A suggested theory is that the stimulation of an autoimmune response could lead to myocarditis and/or pericarditis, as indicated in documented cases [51, 82]. Furthermore, some patients exhibit symptoms of 5-FU toxicity within 72 hours following infusion. This time coincides with a potential delayed sensitivity reaction, given that the plasma half-life of 5-FU is between 12 and 20 minutes. Indeed, reported case exists where corticosteroids showed effectiveness in this condition [83]. Nevertheless, relying solely on the short half-life of 5-FU may not be accurate, given that the drug generates active metabolites that could have an impact [59].

##### 1.5.4.2 Role of Kounis Syndrome

Karabay *et al.* in a case report discuss the possible involvement of Kounis syndrome as another mechanism for 5-FU induced cardiotoxicity [81]. Kounis Syndrome as described by Kounis and Zavras in 1991 is an allergic angina syndrome caused due to release of inflammatory mediator under any hypersensitivity reaction [84]. In terms of Dr. Kounis' definition, the syndrome includes two distinct forms. Type I variation characterized by Coronary artery spasm (which may or may not increase the level of biomarker in the body), is brought on by an allergic reaction in people with healthy coronary arteries who lack any coronary artery disease associated risks. This may trigger coronary vasospasm by disrupting the atherosclerotic plaques established and aggravates as cardiac side effect caused by 5-FU exposure [85].

##### 1.5.4.3 DPYD Gene Mutation

Metabolism of over 80% of the 5-FU metabolic pathway is catalyzed by dihydropyrimidine dehydrogenase (DPD) and insufficient DPD function might be a substantial factor in the exacerbation of cardiotoxicity [86]. DPYD exhibits significant polymorphism, and certain variants such as c.1236G>A/HapB3, c.1679T>G, c.1905+1G>A, and c.2846A>T can result in deficient enzyme activity, increasing the likelihood of severe adverse drug reactions [87]. Variations in the DPD gene's (DPYD) sequence caused by different single-nucleotide polymorphisms (SNPs) might result in a fractional or complete lack of DPD activity, which can be extremely hazardous when combined with fluoropyrimidines [87, 88]. DPD deficiencies significantly increase the likelihood of critical toxicity after fluoropyrimidine treatment, with 60–80% of individuals deficient in DPD experiencing dose-limiting and grave adverse effects that has potential to cause life threatening condition, compared to only 10–20% in those with standard DPD activity [89]. Neutropenia is the predominant toxicity linked to DPD deficiency, as indicated by a study where 55% of patients with decreased DPD activity developed stage IV neutropenia, compared to only 13% of those with normal DPD activity [90]. Similar discrepancies in toxicity were observed in other studies. So, it is safe to assume that individuals possessing these single-nucleotide polymorphisms (SNPs) exhibit an elevated incidence and accelerated onset of severe neutropenia [91]. Saif *et al*., in their case study, reported the first documented instance of Takotsubo Cardiomyopathy (TCM) associated with 5-fluorouracil (5-FU) in a patient with DPYD gene abnormalities. The patient, diagnosed with stage IIIB rectal adenocarcinoma and undergoing intravenous infusion of 5-FU, exhibited clinical manifestations consistent with TCM. Genetic analysis revealed that the patient was heterozygous for the c.85T>C mutation, leading to reduced DPYD enzymatic activity. Nonetheless, the study failed to establish a definitive correlation between 5-FU-induced cardiotoxicity and DPD deficiency, as the evidence supporting this association has thus far been limited to case reports [92]. In another study conducted by Milano *et al*., cardiovascular damage was observed infrequently among patients with clinical DPD deficiency. The researchers reported one case of cardiotoxicity associated with 5-FU treatment out of 19 patients (5%) who had a DPYD polymorphism [86]. However, some research indicates no meaningful correlation between DPYD mutations and the emergence of cardiovascular damage linked to 5-FU [93, 94]. While the exact mechanism of 5-FU-induced cardiotoxicity remains unclear, polymorphic variations in the DPYD gene are hypothesized to be a potential contributing factor; nevertheless, no definitive consensus has been established regarding this association. Importantly, screening for DPD deficiency before treatment, through the identification of common DPYD polymorphisms, plays a crucial role in improving outcomes, especially when combined with personalized dose adjustments [93].

### 1.6 Management of Cardiotoxicity

#### 1.6.1 Diagnosis

Currently, there are no standardized clinical criteria or tests available to specifically detect heart problems associated with fluoropyrimidines [77]. Therefore, it is important for medical professionals to retain a high level of suspicion for cardiac complications in patients undergoing fluoropyrimidine treatment [95]. Cardiotoxicity affiliated with 5-FU can be life-threatening [26], necessitating immediate cessation of chemotherapy upon suspicion to prevent potentially fatal outcomes like ischemia, arrhythmia, or sudden cardiac death [23]. Nevertheless, the preventive use of vasodilatory drugs is not well-supported by data. The pivotal moment is establishing if the cardiac symptoms may be plausibly linked to 5-FU. This presents a conundrum because stopping the medication too soon could jeopardize the patient's chances of recovery, yet continuing it could cause unneeded harm [21].

When fluoropyrimidine-related heart issues are suspected, the initial assessment should include an electrocardiogram (ECG) to check for signs of reduced blood flow to the myocardium, abnormal heart rhythms, and conduction disturbances. Furthermore, assessing levels of cardiac biomarkers aids in identifying injury to myocardial cells. While Troponin levels are commonly checked to assess fluoropyrimidine-related cardiac injury, it is worth noting that patients experiencing coronary vasospasm may not show elevated troponin levels. A study involving 22 individuals receiving 5-FU chemotherapy found no detectable troponin I in any patient throughout the treatment period [96]. However, during a phase II clinical trial involving 22 participants, elevated troponin I levels above the normal range were detected in up to 5% of patients receiving 5-FU [97]. Regarding other cardiac biomarkers, a prospective study with 106 patients undergoing FOLFOX treatment indicated slight rises in NT-proBNP levels during therapy, averaging 28 pmol/L. Those with more pronounced cardiac symptoms generally exhibited higher NT-proBNP levels [23]. However, neither during therapy nor during follow-up, changes in NT-proBNP levels could be used to accurately predict changes in the heart's ejection fraction (EF), as determined by echocardiogram. It is interesting to note that although higher levels of lactic acid, von Willebrand factor, and D-dimer were found in this study, there was no correlation between these changes and the severity of cardiac symptoms. Global longitudinal strain (GLS) or MRI may also be used to transthoracic echocardiography to identify early signs of cardiac dysfunction in patients with elevated cardiac biomarkers but no change in ejection fraction as assessed by the test [77]. Diagnosis approach for 5-FU cardiotoxicity suspect is graphically represented in Fig. (**3**).

#### 1.6.2 Treatment

There is currently no universally accepted standard treatment guidance for cardiotoxicity induced by the use of FPs [11]. The treatment approach is in accordance with the individual's clinical symptoms and the progression of the Cardiovascular condition. The first thing to do when a patient is suspected of having cardiotoxicity is to stop 5-FU or capecitabine medication right away because there is a danger of life-threatening ischemia, arrhythmia, and sudden cardiac death [75]. Effective treatment methods include Trial rechallenge with 5-FU as bolus regimen, using of alternative non-5FU drugs or alternative FU drugs, and changes in treatment modality [21]. Along with it various approaches that are said to be ineffective measures are also followed in adjuvant to the main treatment approach it includes reduction of the dose [98], using drugs like CCB or nitrates as prophylactic treatment [11], and use of Uridine to compete with toxicity associated with FUTP metabolite [99].

##### 1.6.2.1 Trial Rechallenges

Fluoropyrimidine treatment is linked with reappearance of cardiac symptoms when administered repeatedly [100]. Therefore, rechallenge of trial remains controversial and ought to be avoided especially when high incidence of recurrence (90%) and risk of death (13%) is detected [33, 100]. Revascularization and medication rechallenge are administered when a patient's angiography results demonstrate the existence of a substantial blockage that accounts for their symptoms. Addressing the occlusive condition could also enhance the patient's capacity to endure a rechallenge, even if the findings do not adequately clarify the patient's symptoms [101]. Based on a case series of patients experiencing chest discomfort possibly due to coronary vasospasm linked to fluoropyrimidine therapy, Clasen *et al.* have proposed rechallenge guidelines for fluoropyrimidines. They recommend administering sublingual nitroglycerin and short-acting diltiazem as needed during 5-FU infusion, along with pretreatment using extended-release nifedipine and isosorbide mononitrate before initiating 5-FU infusion. They also advise post-treatment with nifedipine and isosorbide mononitrate, with additional nifedipine doses at designated intervals. It is recommended to switch from infusion 5-FU to a bolus regimen whenever possible [102]. This approach of management requires close clinical and continuous ECG monitoring, rechallenge must be promptly discontinued when any signs/ symptoms are detected [5].

##### 1.6.2.2 Pharmacological Intervention for Treatment and Prevention

After the early cessation of fluoropyrimidine administration, the first approach of treatment is done by delivery of nitrates or CCBs to reduce the symptoms and is also been assessed as preventive strategies [11]. Patients showing consistent symptoms of myocardial ischemia through electrocardiograph evidences are generally treated empirically with antianginal medications like CCBs or nitrates, which have successfully relieved symptoms in up to 68% of cases in a retrospective investigation involving 377 patients who FIC [22, 33]. However, there is inadequate data to back up the preventative usage of vasodilatory medications. In a seminal study conducted by Salepci *et al.,* they discovered that pre-treatment with ACE inhibitors provided no benefit and these findings, along with another study, have discouraged significant further research into pretreatment prophylaxis [74, 102]. Alternatively, Ambrosy *et al.* observed that five patients experiencing dyspnea and chest pain after receiving an initial dosage of capecitabine saw a reduction in symptoms when diltiazem was given alongside the FP medication [103]. Furthermore, in a case report nifedipine was found to be useful in reducing 5-FU-induced coronary vasospasm after continuous infusion of 5-FU for stomach cancer [104]. Nitrates have also been used with varying degrees of success. Currently, there are no randomized trials assessing the effectiveness of calcium channel blockers or nitrates in this context. These agents may be considered on a case-by-case basis [11]. Nevertheless, the effect of dose dependent cardiotoxicity related to 5-FU is unclear causing a doubt for the ineffective treatment method of dose reduction [22, 33, 105].

##### 1.6.2.3 Use of Various Non-FU Drugs

Medication like as irinotecan monotherapy or concurrently with another chemotherapeutic drug like oxaliplatin, cetuximab or panitumumab are used in place of 5-FU for patients undergoing treatment for metastatic disease like colorectal cancer (specially RAS/BRAF wild type tumors) in attempt to reduce the cardiotoxicity related with 5-FU [21]. Drugs like Trifluridine-tipiracil, regorafenib and ramucirumab were also preferred as alternative drug to 5-FU in treatment of metastatic disease alone or in combination [105, 106]. Nevertheless, it is difficult for patient with gastrointestinal cancers to make this switch as 5-FU is integral component of the therapeutic approach. In cases of coronary syndrome associated with type I Kounis syndrome, treatment with antihistamines and corticosteroids has demonstrated effective therapeutic outcomes [81, 85].

##### 1.6.2.4 Alternative Treatment Modalities

Use of substitute FU drugs or incorporation of diverse treatment options has additionally been associated to reduce the cardiotoxicity. Patients may consider locally directed treatments like radiofrequency ablation, trans arterial chemoembolization, radioembolization, or surgery if they are not candidates for alternative chemotherapy drugs or if their disease is localized. However, this approach requires careful patient selection and a thorough evaluation of the risk and benefits [21]. Use of medications like as S1, an oral fluoropyrimidine (FP) medicine, has been shown to have antagonistic effects on DPD by slowing the breakdown of 5-FU into FBAL and reducing cardiovascular toxicity. S1 is composed of tegafur and gimeracil, prodrugs of fluorouracil and oteracil potassium, respectively [78, 107]. UFT (Uracil-tegafur) an oral combination drug of tegafur (prodrug of FU) and uracil (agent that decreases FU degradation and increase its concentration) is seen to reduce the incidence of cardiovascular toxicity by 1% [108]. However, these drugs awaits for its approval in USA but is commonly used in Japan for cancers like gastric, colon, craniofacial region, pancreatic *etc*. [21]. US-FDA has approved TAS-102, a FP drug that has lower cardiotoxicity when given to the patient of colorectal cancer [107].

##### 1.6.2.5 Antidote Therapy

Uridine triacetate, a competitive antidote with the 5-FU metabolite fluorouridine triphosphate (FUTP) for incorporation into RNA, may be helpful for patients who fail to respond to stopping fluoropyrimidines and vasodilator therapy [109]. Uridine triacetate is frequently administered as an immediate intervention, typically at a dose of 10 grams every six hours over a span of five days. It's crucial to begin treatment within 96 hours of receiving 5-FU or capecitabine. Uridine triacetate may offer advantages to patients with genetic mutations that increase their susceptibility to fluoropyrimidine hypersensitivity [28]. Although, no such studies has been performed to evaluate its effect in FU induced cardiotoxicity, Uridine triacetate was FDA-approved in 2015 for treating severe, life-threatening toxicity of the cardiovascular and central nervous systems resulting from overdoses of 5-FU or capecitabine [109].

The duration of therapy for FIC is dictated by the symptom’s presentation of the underlying cardiac ailment in each patient. There are no recognized risk ratings for fluoropyrimidine cardiotoxicity, and prospective or randomized controlled trials have not demonstrated the effectiveness of cardioprotective measures [74, 110-112]. Furthermore, DNA screening for mutations in DYPD and TYMS genes holds promise in aiding decisions about the risk *versus* benefit of re-challenging patients who have experienced fluoropyrimidine-related cardiac toxicity. Diagram illustrating the proposed therapy regimen for possible 5-FU cardiotoxicity is represented in Fig. (**4**).

## CONCLUSION

Fluoropyrimidine is still a well-known chemotherapeutic drug to treat various kind of malignancies but the associated cardiotoxicity is yet to be completely understood. Fluoropyrimidine-induced cardiotoxicity, which occurs in 1.2%-18% of patients, significantly increases mortality rates (2.2%-13.3%), particularly with continuous 5-FU infusion. In a 2022 study involving 129 patients with colorectal cancer, 15.5% developed fluoropyrimidine-induced cardiotoxicity (FIC), with the most common symptoms being chest pain and shortness of breath. Notably, only 15% of patients exhibited changes on their electrocardiogram (ECG), including one case of acute myocardial infarction. This emphasizes the need for vigilant clinical monitoring, as ECG abnormalities may not always correspond with the presence of symptoms. Another study observed that the clinical manifestations of cardiac toxicity associated with both 5-FU and capecitabine are similar. Chest pain or angina was reported in approximately 2.27% of patients, with other symptoms including shortness of breath (0.89%), palpitations (0.64%), and hypertension (0.04%). Severe cardiac events, such as heart failure, myocardial infarction, cardiogenic shock, cardiac arrest, and sudden death, are less common but still present a significant risk.

Early symptoms are a major cause of disease burden highlighting the urgent need to address this issue. Cardiotoxicity related to FP can be due to factors such as coronary vasospasm and direct damage to myocardial cells, but further studies are required to fully understand this. Managing affected patients involves determining if 5-FU is the cause of cardiotoxicity, identifying and treating any other cardiac conditions, and deciding whether to continue 5-FU or switch to safer alternatives. Due to the potential severity of these adverse effects, clinicians should be cautious with additional 5-FU doses, consider preventive antianginal treatments, and closely monitor patients, stopping therapy if necessary. Healthcare providers should remain highly alert and incorporate routine cardiovascular monitoring for patients receiving FP chemotherapy. Furthermore, identifying predictive biomarkers, such as specific microRNAs, could improve early diagnosis and allow for more personalized treatment strategies. Randomized clinical trials are needed to establish the best treatment approaches for these patients.

## Figures and Tables

**Fig. (1):**
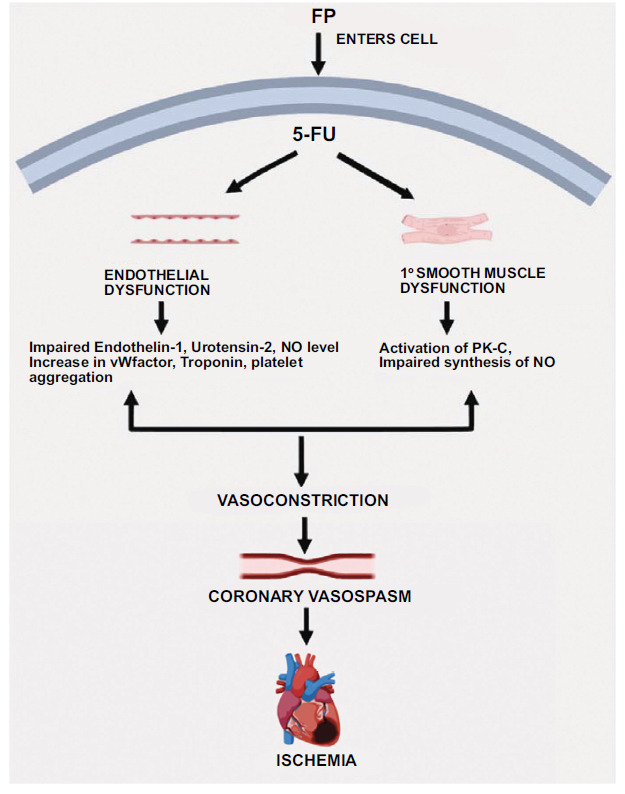
Mechanism of cardiotoxicity due to 5-FU: coronary vasospasm because of direct impairment of the endothelium and primary smooth muscle dysfunction.

**Fig. (2):**
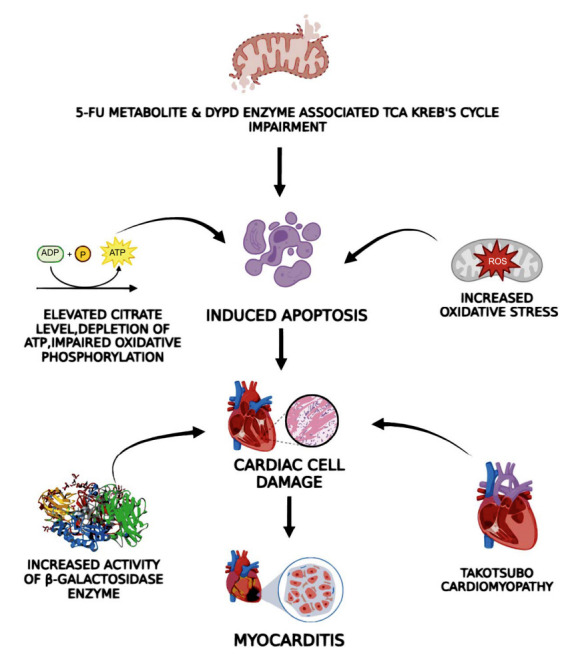
Mechanism of 5-FU induced cardiotoxic event: myocarditis (direct myocardial cell damage) along with various factor contributing to the event.

**Fig. (3):**
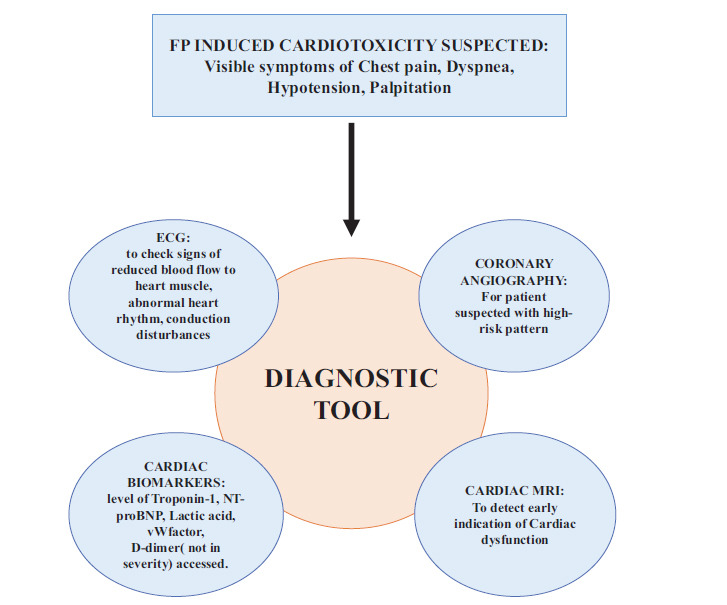
Graphical representation of various diagnostic approach for 5-FU cardiotoxicity suspect.

**Fig. (4):**
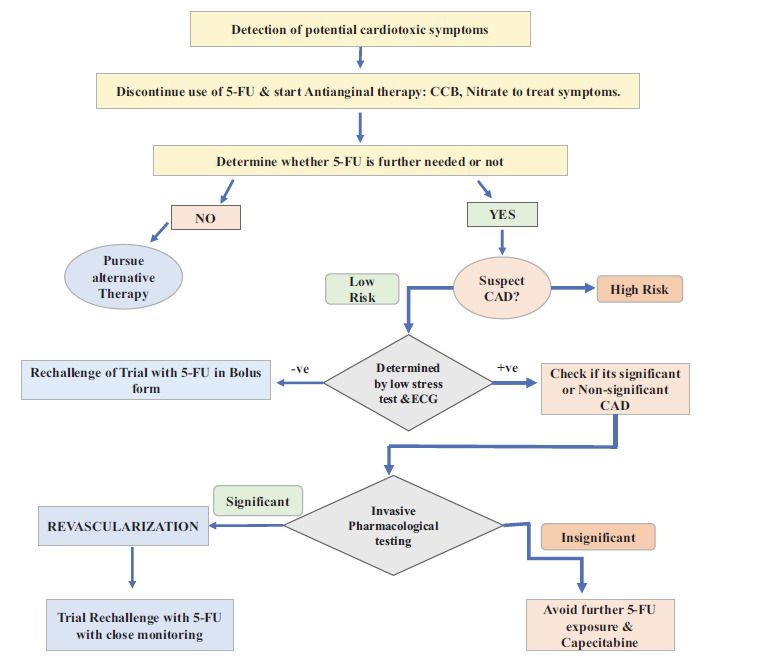
Diagram illustrating the proposed therapy regimen for possible 5-FU cardiotoxicity.

**Table 1:** Demonstrates the incidence rates of cardiotoxicity linked to various fluoropyrimidine treatment regimens used in treating different types of cancer.

**Route of Administration**	**Rate of Incidence**
Bolus intravenous injection	5% [11, 22]
Continuous intravenous infusion	10-18% [11]
FOLFOX regime (oxaliplatin + leucovorin+ 5-FU infusion)	9% [21, 23]
Capecitabin	3-9% [9, 10]
Continuous intravenous injection	3-9% [10]

**Table 2:** Illustrates an overview of research assessing the frequency of cardiotoxicity in patients receiving 5-FU treatment.

**Study**	**Cancer Studied**	**Study Design**	**Sample Population**	**Treatment Type**	**Frequency of Cardiotoxicity**
Jensen *et al.* [23] (2010)	Colorectal	Prospective	106	Continuous 5-FU infusion	5-FU related: 5%
Khan *et al.* [17] (2012)	Not specified	Retrospective	301	5-FU	5-FU related: 19.9%
Lestuzzi *et al.* [24] (2014)	Not specified	Prospective	358	Continuous 5-FU infusion	5-FU related: 10.3% 21(5.9%) at rest & 16(7%) under effort.
Polk *et al.* [25] (2016)	Breast	Retrospective	452	Capecitabine	Capecitabine related: 4.9-5%
Kwakman *et al.* [26] (2017)	Colorectal	Retrospective	2133	Capecitabine	Capecitabine related: 5.9%
Peng *et al.* [27] (2018)	Not specified	Prospective	527	5-FU (196 patient) Capecitabine (331 patient)	5-FU related: 25% Capecitabine related:33.8%
Raber *et al.* [28] (2019)	Not specified	Retrospective Case-Control Study	177	5-FU or Capecitabine	5-FU related: 4.2% Capecitabine related: 5.3%
Jin *et al.* [29] (2019)	Gastro-intestinal	Retrospective	129	5-FU based	5-FU related: 29.5%
Dhyl-Polk *et al.* [30] (2020)	Colorectal	Retrospective	2236	5-FU or Capecitabine	5-FU related:5% Capecitabine related: 4% (overall: 5.2%)
Osterlund *et al.* [31] (2022)	Not specified	Retrospective observational cohort study	200	Capecitabine or Continuous infusion/bolus 5-FU	Capecitabine: 85% (in 170 patient) Continuous infusion 5-FU: 12% (in 22 patient) Bolus 5-FU: 4% (in 8 patient)
Lestuzzi *et al.* [32] (2022)	Not specified	Prospective	192	Capecitabine	Capecitabine related: 16.7% (stress aggravated)

## LIST OF ABBREVIATIONS

BMI: Body Mass Index
CVD: Cardiovascular Diseases
ECGs: Electrocardiograms
FIC: Fluoropyrimidine-induced Cardiotoxicity
FP: Fluoropyrimidine
MI: Myocardial Infarction
TS: Thymidylate Synthase
